# Qualitative and quantitative detection of surgical pathogenic microorganisms *Escherichia coli* and *Staphylococcus aureus* based on ddPCR system

**DOI:** 10.1038/s41598-021-87824-5

**Published:** 2021-04-22

**Authors:** Tiantian Zhang, Zhiqiang Niu, Feng Wu, Zongkun Chen, Jun Xu, Kewei Jiang, Zhiyong Lai

**Affiliations:** 1 Assisted Reproduction Center, The First Hospital of Shanxi Medical University, Yingze District, No. 85 Jiefang South Road, Taiyuan, 030001 People’s Republic of China; 2 Shanxi Medical University, Yingze District, No. 56 Xinjian South Road, Taiyuan, Shanxi 030001 People’s Republic of China; 3 Department of General Surgery, The First Hospital of Shanxi Medical University, Yingze District, No. 85 Jiefang South Road, Taiyuan, 030001 People’s Republic of China; 4 Department of Gastroenterological Surgery, Laboratory of Surgical Oncology, Beijing Key Laboratory of Colorectal Cancer Diagnosis and Treatment Research, Peking University People’s Hospital, No.11 Xizhimen South Street, Xicheng District, Beijing, 100044 People’s Republic of China

**Keywords:** Biochemistry, Biotechnology

## Abstract

Bacterial culture and drug susceptibility testing are used to identify pathogen infections. Nevertheless, the process requires several days from collection to the identification of bacterial species and drug-resistance patterns. The digital PCR system is a rapidly developing quantitative detection technology widely applied to molecular diagnosis, including copy number variations, single nucleotide variant analysis, cancer biomarker discovery, and pathogen identification. This study aimed to use a droplet digital PCR system to identify bacteria in blood samples and explore its ability to identify pathogen in bacteremia. Then, we designed primers and probes of SWG-9 and COA gene for *E. coli* and *S. aureus* to identify in blood samples with the ddPCR system. The system had demonstrated extremely high detection accuracy in blood samples, and the detection rate of *E. coli* was 13.1–21.4%, and that of *S. aureus* was 50–88.3%. Finally, blood samples containing both *E. coli* and *S. aureus* were tested to evaluate further the accuracy and applicability of this method, indicating the detection rates range from 18.1% to 97%. The ddPCR system is highly promising as a qualitatively and quantitatively screening method for rapidly detecting pathogen.

## Introduction

Despite economic development, with health systems in various countries worldwide continuously improving, infectious diseases are still one of the main threats to human health. The 2018 Global Burden of Disease Study reported that 7.069 million people died from infectious diseases worldwide, accounting for 12.6% of the global deaths, and the mortality rate was 97/100000^1^. Infectious diseases remain a significant public health problem. Early diagnosis and rational application of antibiotics are essential for significance for the management of infectious diseases. At present, the clinical use of antibiotics depends on empirical treatment. Also, the over-use of antibiotics increases the resistance of bacteria and drug-induced diseases and consumes substantial health care resources^2^. Studies have reported that the annual cost of treatment for infectious diseases was about $1.5–2 billion^3^. One of the reasons was that the pathogenic microorganisms could not be quickly and accurately identified in the early stage of infections, leading to irrational use of antibiotics and other medical treatment measures.

The most common pathogenic bacteria in general surgery departments are *Escherichia coli, Staphylococcus aureus, Pseudomonas aeruginosa, Klebsiella pneumoniae*, and *Acinetobacter baumannii*^4^. Traditional bacterial detection and identification methods are based on bacterial culture and biochemical identification, and testing usually requires several days, even to one week. As a gold standard, the diagnosis of bacterial infectious diseases often relies on the specific detection of pathogenic bacteria. The use of antibiotics must be based on the bacterial culture results and drug sensitivity test results in clinical diagnosis and treatment. However, due to detection time, bacterial culture growth characteristics, non-pathogenic bacteria contamination, and improper material selection, the test results in actual work may generate false negatives or false positives. Incorrect identification results not only in delayed treatment, wasting medical resources, but in mistaken diagnosis and treatment. For these reasons, rapid, accurate identification of pathogenic microorganisms has become an intense focus of research.

In recent years, molecular biology techniques have been applied to detect pathogenic microorganisms. They have attracted attention due to their high specificity, lower time-requirement, and reduced incidence of cross-infection. These techniques include Enzyme-Linked Immunosorbent Assay^2^, Polymerase Chain Reaction^5^ and Gene microarray chips^6^. To date, Polymerase Chain Reaction technology has been widely used in many research fields because of its simple, intuitive, economical, and rapid detection characteristics^7^. Digital polymerase chain reaction (dPCR) was first proposed by Kenneth Kinzler and Bert Vogelstein in 1999. This is a PCR technology that truly achieves absolute quantification after qPCR technology^8^. Droplet digital PCR (ddPCR) is a new technology that enables absolute quantification of nucleic acids with high analytical sensitivity and precision. ddPCR splits PCR reagents into tens of thousands of nanoliter or picoliter partitions by a microfluidic chip so that each droplet contains 0 or 1 DNA template. After PCR amplification and fluorescence detection, the target nucleic acids are calculated from the number of positive and negative droplets by Poisson statistics^9^. Compared with traditional qPCR, digital PCR has higher sensitivity, specificity, and accuracy. It plays an essential role in many fields, including early diagnosis of tumor markers^10^, gene expression product analysis^11^, food safety testing^12^, pathogenic microorganism testing^13^, common genetic disease testing, and non-invasive prenatal diagnosis^14^.

In the present study, we designed primers and probes of SWG-9 and COA gene for *E. coli* and *S. aureus*. Then, ddPCR was applied to test the advantage and reliability for identifying *E. coli* and *S. aureus* in simulated bacteremia blood samples to provide the theoretical basis for subsequent clinical sample testing.

## Results

### Assay design and identification of bacterial strains using the ddPCR system

To examine the method's dynamic range and detection limit, serial dilutions of *E. coli* and *S. aureus* DNAs were subjected to ddPCR. Bacterial DNA was quantified using Qubit 3.0, and the estimated copy number was calculated. Then, we diluted the bacterial DNA templates in different concentrations.

**Figure 1:**
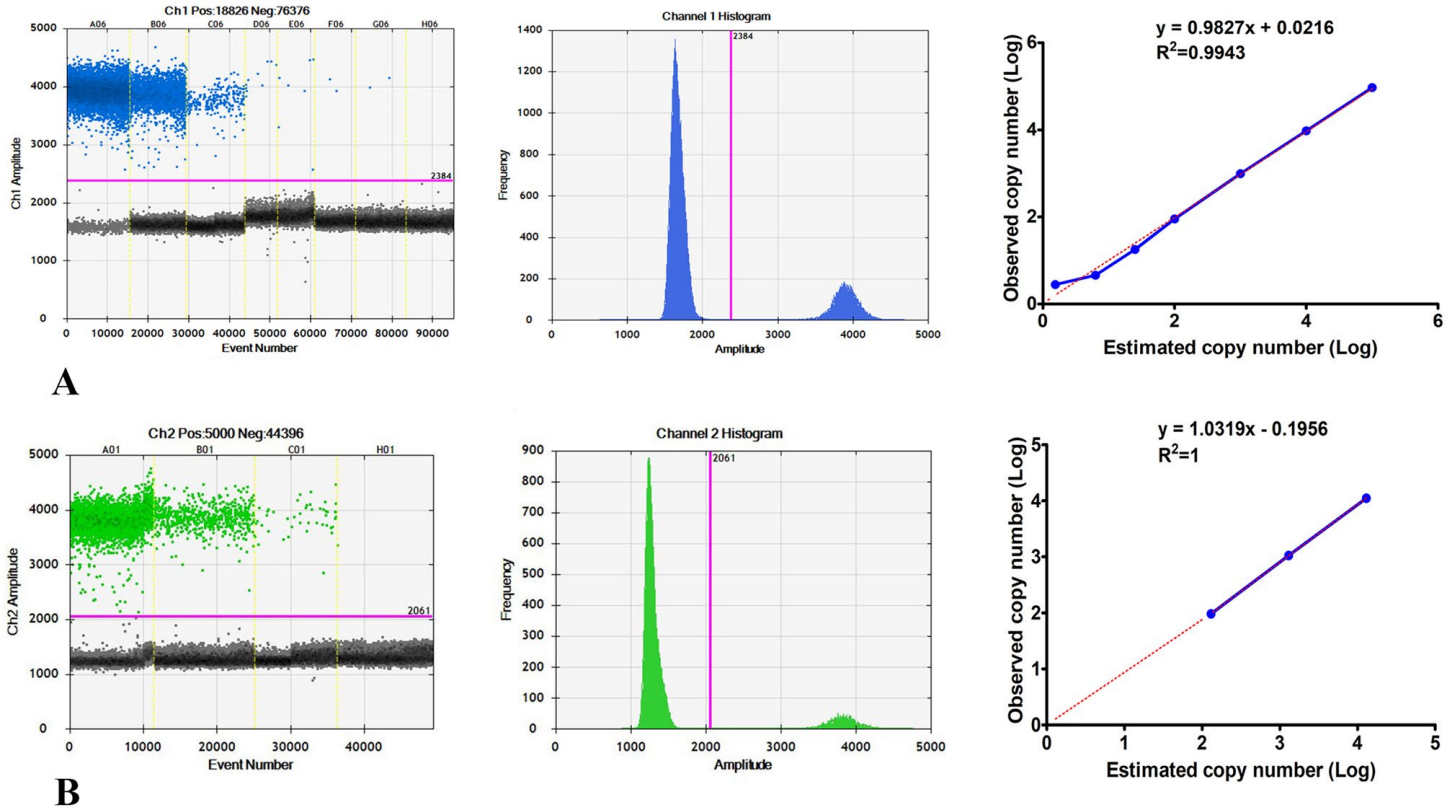
The dynamic range of the ddPCR-based assay. Detecting the bacterial nucleic acid by ddPCR system, (**A**) The 1D droplet spots of FAM fluorescence amplitude for eight detection sites for E. coli ATCC25922 (from A06 to H06: the estimated copy numbers of nucleic acid template were 10^5^, 10^4^, 10^3^, 10^2^, 25, 6.25, 1.56, and NC) were performed for each detection site. Linear fitting lines are shown on the right (**B**) The 1D droplet spots of VIC fluorescence amplitude for four detection sites for *Staphylococcus aureus* ATCC25923 (from A01 to H01: the estimated copy numbers of nucleic acid template were 10^4^, 10^3^, 10^2^, and NC) were performed for each detection site. Linear fitting lines are shown on the right.

The ddPCR-based bacterial strains detection workflow mainly consisted of five steps: sample preparation, preparation of reaction mixture, droplet generation, PCR amplification, and fluorescence detection and data analysis. For strains of bacteria, and the number of bacterial nucleic acids can be detected simultaneously. For NC, all experiments performed in this study showed no positive signal in either the FAM or the VIC channel, indicating no contamination in the ddPCR system. To examine the dynamic range of the method, serial dilutions of *E. coli* or *S. aureus* were quantified by ddPCR, and the results of three replicates were analyzed (Fig. 1, S3; Tables 1, 2, S11, S12).

**Table 1:** Dynamic range of the ddPCR-based assay. Detecting the E. coli ATCC25922 nucleic acid template by ddPCR system.

Counts of bacterial nucleic acid template	430 pg	43 pg	4.3 pg	430 fg	107.5 fg	26.9 fg	6.7 fg	0
Theoretical copy number	100,000	10,000	1000	100	25	6.25	1.56	0
Logarithmic transformation	5	4	3	2	1.40	0.80	0.19	-
Actual number of copies	93,200	9460	988	90	18	4.6	2.8	0
Logarithmic transformation	4.969	3.976	2.994	1.954	1.255	0.663	0.447	–

**Table 2:** Dynamic range of the ddPCR-based assay. Detecting the Staphylococcus aureus ATCC25923 nucleic acid template by ddPCR system.

Counts of bacterial nucleic acid template	31 pg	3.1 pg	310 fg	0
Theoretical copy number	13,000	1300	130	0
Logarithmic transformation	4.1139	3.1139	2.1139	–
Actual number of copies	11,120	1058	96	0
Logarithmic transformation	4.0461	3.0245	1.9823	–

The ddPCR system identifies various *E. coli* ATCC25922 nucleic acid (Fig. 1A, corresponding to A05 to H05). The ddPCR signals were linear over the range from 0 to 10^5^ copies/μL with R^2^ = 0.9995 for all sites. The actual copy number of bacteria from A05 to E05 measured using the ddPCR system (Fig. 1A Table 1). The ddPCR system identified various *S. aureus* ATCC25923 nucleic acid (Fig. 1B, corresponding to A01–H01), and the measured number of bacterial nucleic acid templates were very close to the theoretical value. The ddPCR signals were linear over the range from 0 to 10^4^ copies/μL with R^2^ = 1 for all sites. The actual copy number of bacteria nucleic acid templates from A01 to C01 measured using the ddPCR system (Fig. 1 B, Table 2).

We compared the lowest detection limit of ddPCR and real-time quantitative PCR in different numbers of bacterial nucleic acid templates. When the number of nucleic acid templates is less than 100, the real-time quantitative PCR system cannot produce a useful amplification curve (Figure S1, S2; Table S8–S10).

### The specificity of ddPCR for simultaneously identified two bacterial strains

**Figure 2:**
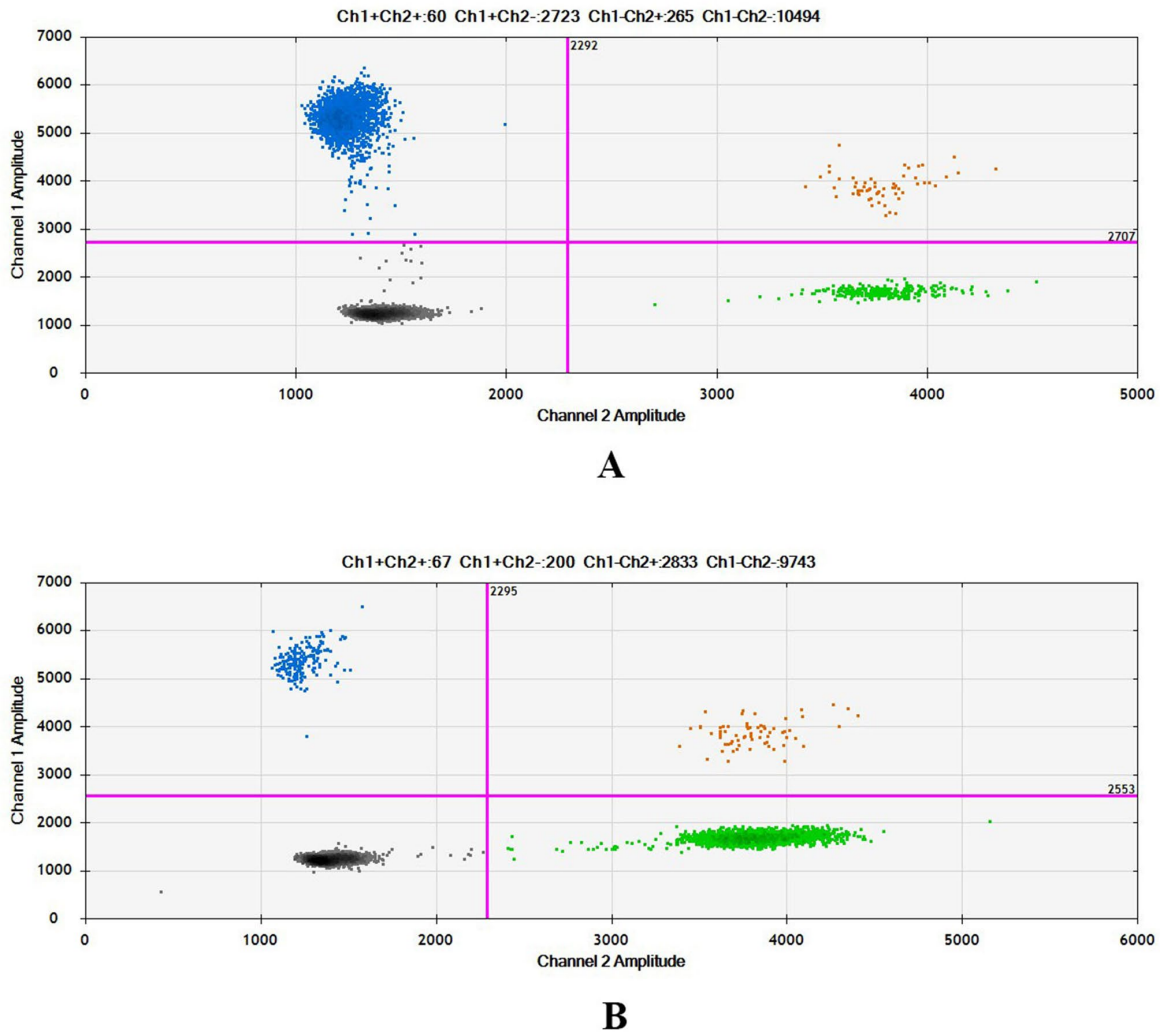
Quantitative results of the ddPCR system simultaneously detecting two bacterial strains. The 2D drop plots of *Escherichia coli* ATCC25922 and *Staphylococcus aureus* ATCC25923 by ddPCR system. (**A**) bacteria counts of *Escherichia coli*: bacteria counts of *Staphylococcus aureus* = 10:1 ( 6500:650 ), (**B**) bacteria counts of E. coli: bacteria counts of *Staphylococcus aureus* = 1:10 (650:6500).

To validate the specificity of this method, two groups of ddPCR amplification experiments, including *E. coli* nucleic acid and *S. aureus* nucleic acid, were performed in triplicates for each detection site. In the two sets of experiments, the actual DNA copy numbers of ATCC25922 and ATCC25923 were very close to the theoretical values, and there was almost no interference between them. The detection rate of bacteria was as high as 80% or even 90% (Fig. 2, Tables 3 and 4).

**Table 3:** Quantitative results of the ddPCR system simultaneously detecting two bacterial strains (bacteria counts of E. coli: bacteria counts of Staphylococcus aureus = 10:1).

Bacterial strain	ATCC25922	ATCC25923
Theoretical value	6500	650
Measured value	5420	572
Extraction rate(%)	83.4	88

**Table 4:** Quantitative results of the ddPCR system simultaneously detecting two bacterial strains (bacteria counts of E. coli∶bacteria counts of Staphylococcus aureus = 1:10).

Bacterial strain	ATCC25922	ATCC25923
Theoretical value	650	6500
Measured value	494	6000
Extraction rate(%)	76	92.3

### Bacteria count

**Figure 3:**
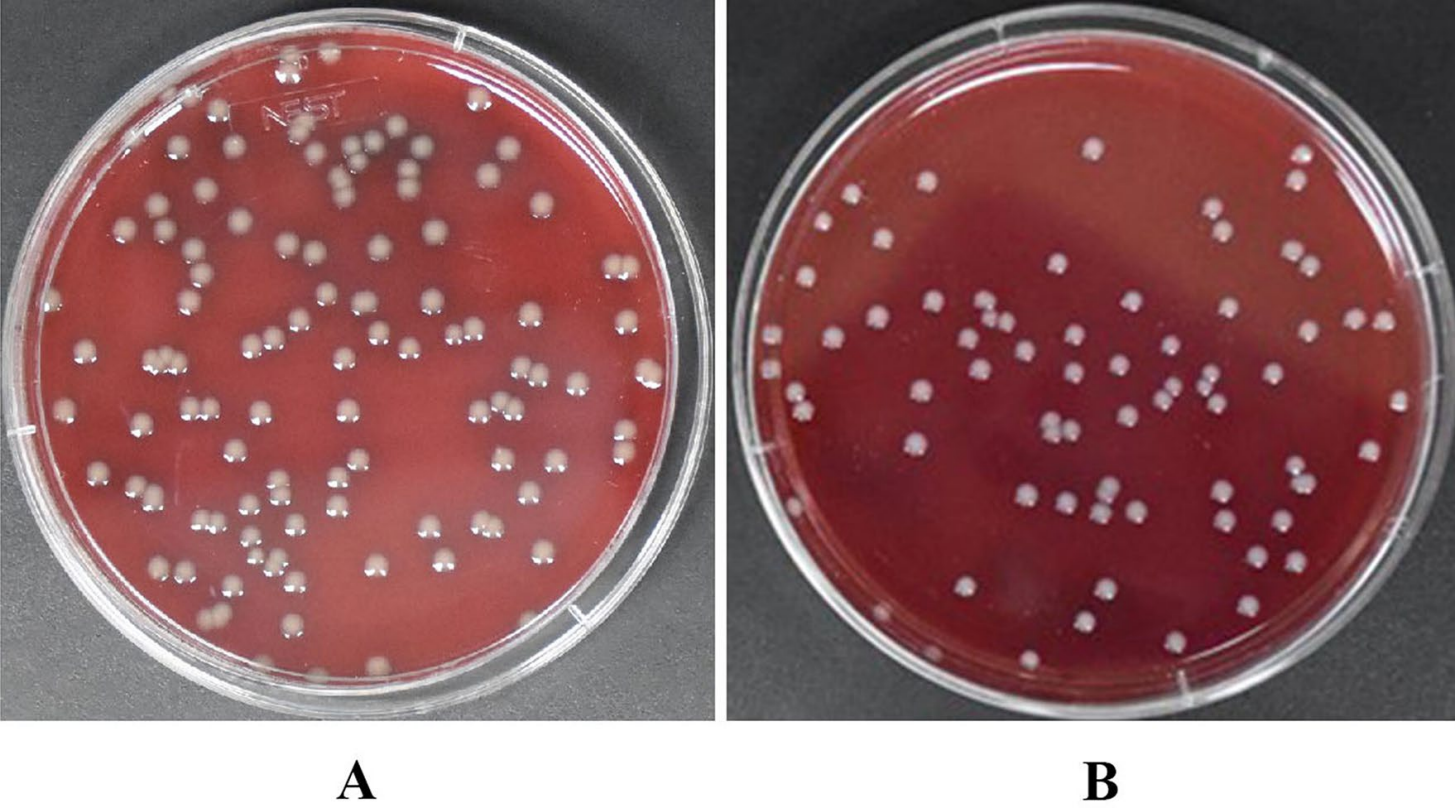
Bacterial culture and bacterial count. (**A**) colony number of *Escherichia coli* ATCC25922 is 107, (**B**) colony number of *Staphylococcus aureus* ATCC25923 is 70.

We added *E. coli* and *S. aureus* to 50 ml of Luria–Bertani bacterial culture medium and placed it in an orbital shaker at 37 °C overnight. We then diluted the bacterial solutions 10^6^ times and performed a rolling ball scratching board. We calculated the number of colonies in the culture medium and found that the number of colonies of *E. coli* ATCC25922 was 107, and the number of colonies of *S. aureus* ATCC25923 was 70 (Fig. 3). Conversion is made according to the bacterial solution's dilution factor. The concentration of the *E. coli* ATCC25922 bacterial solution was 1.25 × 10^9^ cells/ml, and the concentration of *S. aureus* ATCC25923 bacterial solution was 6.7 × 10^8^ cells/ml.

### Detection of simulated bacteria using the ddPCR system

**Figure 4:**
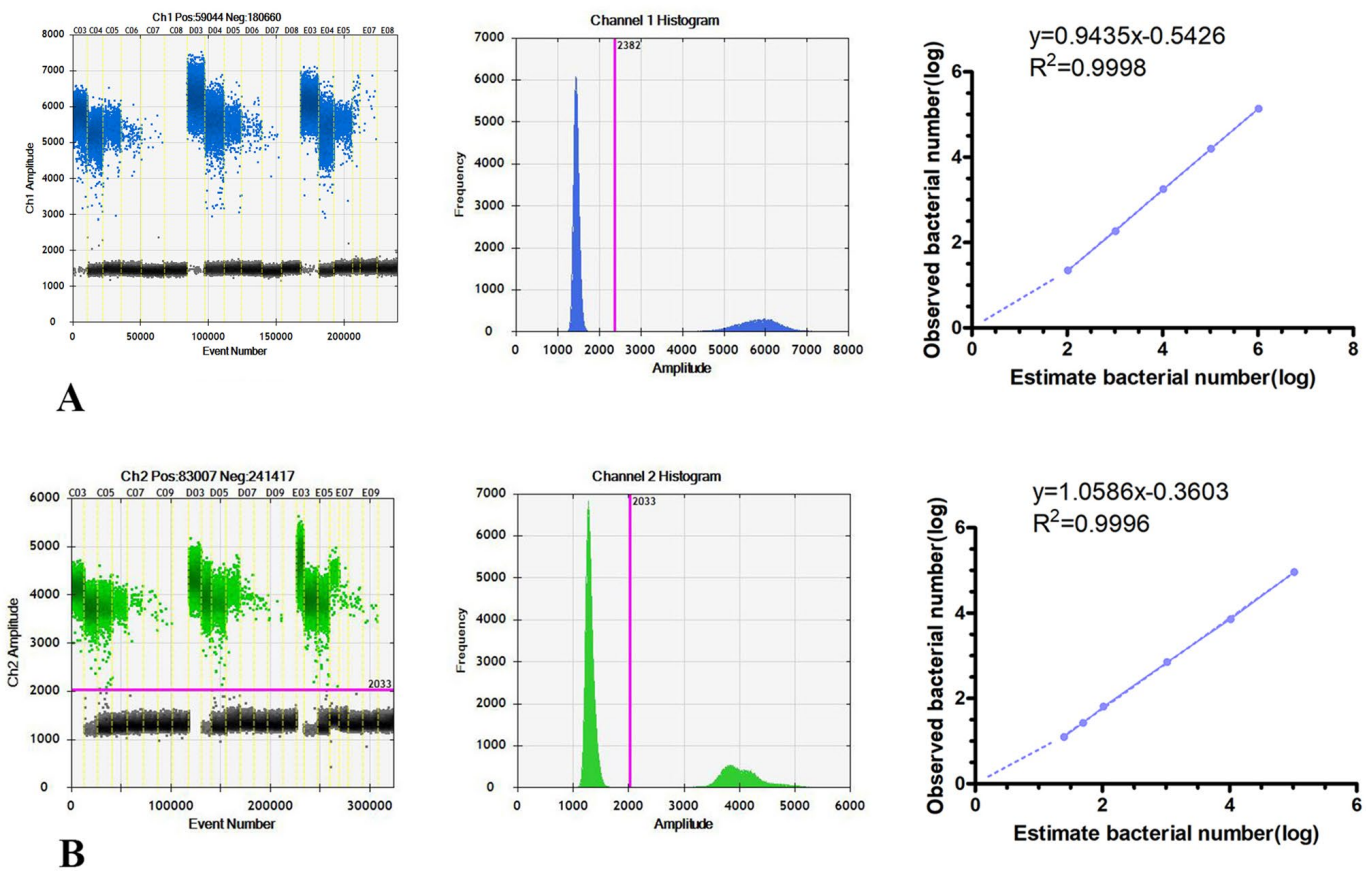
The dynamic range of the ddPCR-based assay for detection of a simulated bacteremia blood sample. (**A**) The 1D droplet spots of FAM fluorescence amplitude for six detection sites for *Escherichia coli* ATCC25922 (from C03-C08, D03-D08, E03-E08: the estimated bacterial counts were 1.04 × 10^6^, 1.04 × 10^5^, 1.04 × 10^4^, 1.04 × 10^3^, 1.04 × 10^2^, and NC ) were performed for each detection site. Linear fitting lines are shown on the right. (**B**) The 1D droplet spots of VIC fluorescence amplitude for eight detection sites for *Staphylococcus aureus* ATCC25923 (from C03-C10, D03-D10, E03-E10: the estimated bacterial counts were 1.04 × 10^6^, 1.04 × 10^5^, 1.04 × 10^4^, 1.04 × 10^3^, 1.04 × 10^2^, 50, 25 and NC) were performed for each detection site. Linear fitting lines are shown on the right. Error bars represent the standard deviation of three replicate samples at each target bacterial count.

We used the ddPCR system to identify bacterial nucleic acid extracted from the simulated bacteremia blood samples. We counted the number of bacteria by counting the number of nucleic acid templates. The number of bacteria detected using the ddPCR system and the theoretical number of bacteria are listed in Tables 5 and 6. We took the average number of bacteria detected in the three groups and calculated the logarithm of the average number of bacteria to perform fitting analysis (Fig. 4, ATCC25922: *R*^2^ = 0.9998, ATCC25923: *R*^2^ = 0.9996). The *E. coli* ATCC25922 nucleic acid detection rate in simulated bacteremia blood samples was 13.1–21.4%, and the *S. aureus* ATCC25923 nucleic acid detection rate in simulated bacteremia blood samples was 50–88.3%.

**Table 5:** Detecting E. coli ATCC25922 nucleic acid extracted from the simulated bacteremia blood sample.

Counts of bacteria	1,040,000	104,000	10,400	1040	104	0
Parallel group 1	136,400	16,460	1924	188	16.8	0
Parallel group 2	137,400	15,680	1740	174	24	0
Parallel group 3	135,800	15,300	1704	192	26	0
Average	136,533.3	15,813.3	1789.3	184.7	22.3	0

**Table 6:** Detecting Staphylococcus aureus ATCC25923 nucleic acid extracted from the simulated bacteremia blood sample.

Counts of bacteria	104,000	10,400	1040	104	50	25	0
Parallel group 1	88,800	7000	730	62	20	12.8	0
Parallel group 2	92,400	7320	694	72	28	11	0
Parallel group 3	94,400	7200	706	62	32	13.6	0
Average	91,866.7	7173.3	710	65.3	26.7	12.5	0

**Figure 5:**
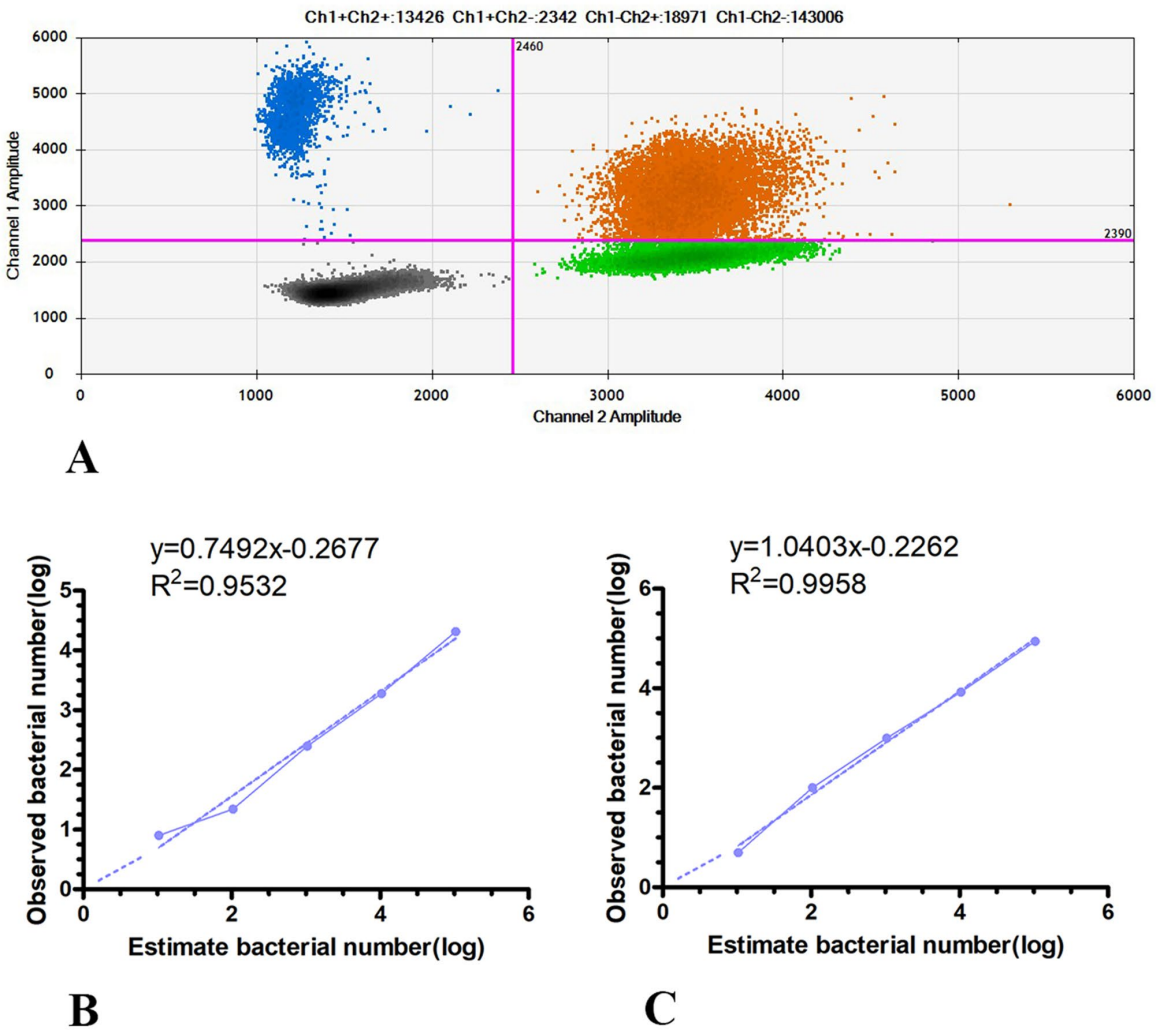
Quantitative results of the ddPCR system simultaneously detecting simulated bacteremia blood sample with *Escherichia coli* ATCC25922 and *Staphylococcus aureus* ATCC25923. (**A**) The 2D drop plots of *Escherichia coli* ATCC25922 and *Staphylococcus aureus* ATCC25923 by ddPCR system. (**B**) The linear fitting curves of *Escherichia coli* ATCC25922 are shown on the left. (**C**) The linear fitting curves of *Staphylococcus aureus* ATCC25923 were shown on the right.

We used the ddPCR system to measure bacterial nucleic acid extracted from the simulated bacteremia blood samples contaminated by *E. coli* and *S. aureus* simultaneously (Table 7 and 8). We took the logarithm of the measured number of bacteria to make a fitting curve (Fig. 5, ATCC25922: R^2^ = 0.9532, ATCC25923: R^2^ = 0.9958). The bacterial nucleic acid detection rate of *E. coli* ATCC25922 was 18.1–77%, and that of *S. aureus* ATCC25923 was 44.9–97%.

$${\text{Detection\,rate}}= {\frac{\text{The\,number\,of\,DNA\,templates\,actually\,tested}} {\text{The\,number\,of\,bacteria}}}\times 100\%$$

**Table 7:** Detecting E. coli ATCC25922 nucleic acid extracted from simulated bacteremia blood samples contaminated by Escherichia coli and Staphylococcus aureus.

Counts of bacteria	104,000	10,400	1040	104	10.4	0
Parallel group 1	20,180	1960	242	36	10	0
Parallel group 2	21,100	1960	248	14	6	0
Parallel group 3	20,440	1740	258	16	8	0
Average	20,573	1887	249	22	8	0

**Table 8:** Detecting Staphylococcus aureus ATCC25923 nucleic acid extracted from simulated bacteremia blood samples contaminated by Escherichia coli and Staphylococcus aureus.

Counts of bacteria	104,000	10,400	1040	104	10.4	0
Parallel group 1	89,000	8380	932	116	10	0
Parallel group 2	87,400	8580	1010	106	0	0
Parallel group 3	85,600	8160	1026	80	4	0
Average	87,333	8373	989	101	5	0

## Discussion

The specific genes of bacterial strains were selected according to the GenBank database. Nandakafle and Brozel first discovered the SWG-9 UIDA gene in 2015. The majority of *E. coli* strains carry the SWG-9 gene, which contains 566 bases and encodes a protein product containing 188 amino acids. So we designed a primer and MGB probe for this gene to detect *E. coli*. Also, Most *S. aureus* carries the *Staphylococcus* coagulase gene, which can encode protein products of 647 amino acids, and this gene plays a vital role in the pathogenic process of *S. aureus*^15^. Therefore, we selected the conserved sequence of this gene in the experiment to design PCR primers and probes to identify strains.

Furthermore, in this study, the sensitivity and accuracy of the ddPCR system have obvious advantages compared with real-time fluorescent quantitative PCR. The ddPCR system retains high sensitivity and accuracy when simultaneously detecting two pathogenic microorganisms. Also, the ddPCR system performs absolute quantification without the need for a standard curve. The detection rate of simulated bacteremia blood samples using the ddPCR system is lower than that of purified bacteria nucleic acid samples, mainly because of the bacterial nucleic acid extraction rate in simulated bacteremia blood samples. When the bacterial nucleic acid is extracted, human blood, EDTA in the anticoagulation tube, whether the cell walls are easily digested and cleaved by enzymes, and the elution and enrichment of nucleic acid, various operating processes and factors have a negative impact during the bacterial nucleic acid extraction. Even so, the rate of extracting bacterial nucleic acid from bacterial blood samples by the ddPCR system was between 15 and 80%.

We found that when the concentration of bacteria in the blood was greater than ten cells/ml, the ddPCR system detected bacteria stably. At present, clinical laboratories determine whether bacteremia is present based on the blood culture instruments reporting positive, and the reporting time is about 6–18 h. Generally speaking, it takes about three days from the collection of blood samples to the completion of bacterial culture and antibiotic sensitivity testing. Suppose we design unique probes and primers for specific bacterial and drug-resistance genes. In that case, the ddPCR system can identify bacterial species and drug-resistance genes simultaneously, with detection times less than 4 h. Therefore, our research suggests that the ddPCR technical platform is superior for the identification of pathogenic microorganisms. The ddPCR system accurately and effectively detect pathogenic microorganisms, allowing for more targeted antibiotic therapy and minimizing misuse of antibiotics, drug toxicity, and drug resistance, all of which could reduce economic burdens on patients.

Currently, the coronavirus (COVID-19) epidemic is spreading all over the world. The pathogenic microorganism of this pneumonia has been identified as a new coronavirus (Severe Acute Respiratory Syndrome Coronavirus 2, SARS-CoV-2). In acute respiratory infections, reverse transcription-polymerase chain reaction is usually used to detect pathogenic viruses from respiratory secretions in nucleic acid testing. It is the "gold standard" for the diagnosis of related cases^16^. However, the RT-PCR test result can also produce false negatives^17^. The reasons are the time of specimen collection, the quality or type of specimen, the transportation of the specimen, the ability of the virus to mutate, and the low sensitivity of RT-PCR and polymerase chain reaction inhibition^18–20^. Therefore, an efficient, fast, and accurate laboratory diagnosis technology is the key to ensuring early diagnosis, timely treatment, and preventing the development of the epidemic. Based on its advantages, digital PCR provides a new, efficient, and accurate detection method for pathogenic microorganisms such as viruses and bacteria detection.

## Methods

### Bacterial strains, experimental reagents, and instruments

The bacterial strains used were provided by the Laboratory of Peking University People's Hospital (Table S1), and the reagents and instruments used were provided by the laboratory of Tsinghua University (Tables S2 and S3).

### Extraction of bacterial nucleic acid

The bacterial strains were propagated and cultured in a 50 ml Luria–Bertani bacterial culture medium. A total of four strains of bacteria were used in the study, *S. aureus* ATCC25923, *S. aureus* ATCC29213, *E. coli* ATCC25922, and a strain of *E. coli* named *E. coli*-clin extracted from a patient. According to the methods suggested in the manufacturer's instructions, nucleic acids were extracted from the four bacteria using the QIAamp cador Pathogen Mini Kit (QIAGEN, Germany). We used Nanodrop to measure the extracted nucleic acid concentrations and stored samples at –20 °C.

### Design of primers and probes

The specific genes of bacterial strains were selected according to the GenBank database. The specific genes of *E. coli* and *S. aureus* were SWG-9 and coagulase COA gene, respectively. Primers and probes were designed for conserved regions in SWG-9 and coagulase COA gene sequences (Table S4 and S5). We used Oligo 7.0 and Primer Express 3.0 software to design the primers and probes for the nucleic acid sequence containing the site to be detected. The sequences were delivered to Thermo Fisher Scientific (CN) to synthesize the primers and probes. The 5 'end of the *E. coli* probe was labeled with a 6-FAM fluorescent molecule, the 5′ end of the *S. aureus* probe was labeled with VIC fluorescent molecule.

### Workflow of the ddPCR system

According to the manufacturer's instructions, we carried out ddPCR using the QX200 ddPCR system (Bio-Rad, CA). The QX200 ddPCR system (Bio-Rad, CA) consists of four steps: (1) preparation of the reaction mixture using a ddPCR amplification volume 20 µL (Table S6); (2) droplet generation; (3) ddPCR amplification (Table S7); and (4) droplet reading and analysis of results using QuantaSoft 3.0 software.

### Detection of bacterial strains using a real-time quantitative PCR system

The real-time quantitative PCR system with the MGB probe system was used to quantify the gradient-diluted nucleic acid of *E. coli* standard strain (ATCC25922) to determine the sensitivity range of the two real-time quantitative PCR. The template dilution gradient (copies/20 µL) of ATCC25922 was 10^6^, 10^5^, 10^4^, 10^3^, 10^2^, 10^1^, 1, and 0. Each group of experiments was repeated three times in parallel.

### Detection of bacterial strains by ddPCR system

We used the ddPCR system to quantify the gradient-diluted nucleic acid of *E. coli* (ATCC25922) to determine the sensitivity range of the ddPCR system. The template dilution gradient (copies/20 μl) of ATCC25922 were 10^5^, 10^4^, 10^3^, 10^2^, 25, 6, and 0. Each group of experiments was repeated three times in parallel.

### The ddPCR system simultaneously detects two bacterial strains

Various numbers of DNA templates, specific primers, and probes (ATCC25922: 5′-FAM/3′MGB, ATCC25923: 5′-VIC/3′MGB) of the two bacterial strains were added to a ddPCR reaction system (Group A, bacteria counts of *E. coli*: bacteria counts of *S. aureus* = 10:1, Group B, bacteria counts of *E. coli*: bacteria counts of *S. aureus* = 1:10). We counted DNA template numbers of *E. coli* and *S. aureus* and calculated the detection rate of the two bacteria.

### Detection of blood samples of a simulated bacteremia blood sample using the ddPCR system

The blood samples were obtained from the Department of Gastroenterological Surgery, Peking University People’s Hospital. All of the blood sample donors included in this study provided their informed consent. The study was approved by the Human Ethics Committee of the Peking University People’s Hospital (China). The blood collection tube was pretreated with EDTA anticoagulation. Three simulations of bacteremia blood samples were prepared; Group A: 100 μL of the serially-diluted suspensions of *E. coli* (ATCC25922) were injected into 400 μL of blood treated with EDTA. Group B: 100 μL of the serially-diluted suspension of the *S. aureus* strain (ATCC25923) was injected into the 400 μL blood treated with EDTA. Group C: 100 µL of the ATCC25922 and ATCC25923 suspensions were diluted into gradients, then added to the blood samples with 400 µL EDTA. Extraction of nucleic acid from bacterial blood specimens was performed as follows: nucleic acid from bacterial blood specimens was extracted using a bacterial nucleic acid kit (Qiagen, Germany). We used 50 µl of solution buffer to elute DNA from the column and stored it at 80 °C. The extracted nucleic acid was detected using the ddPCR system. Repeat the test three times in parallel.

### Statistical analysis

The detection results were statistically analyzed using SPSS 22.0, GraphPad Prism 5, and OriginPro 2016 software, and the linear dynamic range of the method was measured by R^2^ value.

### Ethics approval and consent to participate

The present study was approved by the Human Ethics Committee of the Peking University People’s Hospital (China) and written informed consent was obtained from all subjects. Methods were carried out in “accordance” with the approved guidelines.

## Supplementary Information

Supplementary Figure S1.

Supplementary Figure S2.

Supplementary Figure S3.

Supplementary Figure Legends

Supplementary Tables
