# Supplementary figures and images for: Qualitative and quantitative detection of surgical pathogenic microorganisms Escherichia coli and Staphylococcus aureus based on ddPCR system
Source: Sci Rep. 2021 Apr 22;11:8771. doi: 10.1038/s41598-021-87824-5 (PMC8062461; doi:10.1038/s41598-021-87824-5)

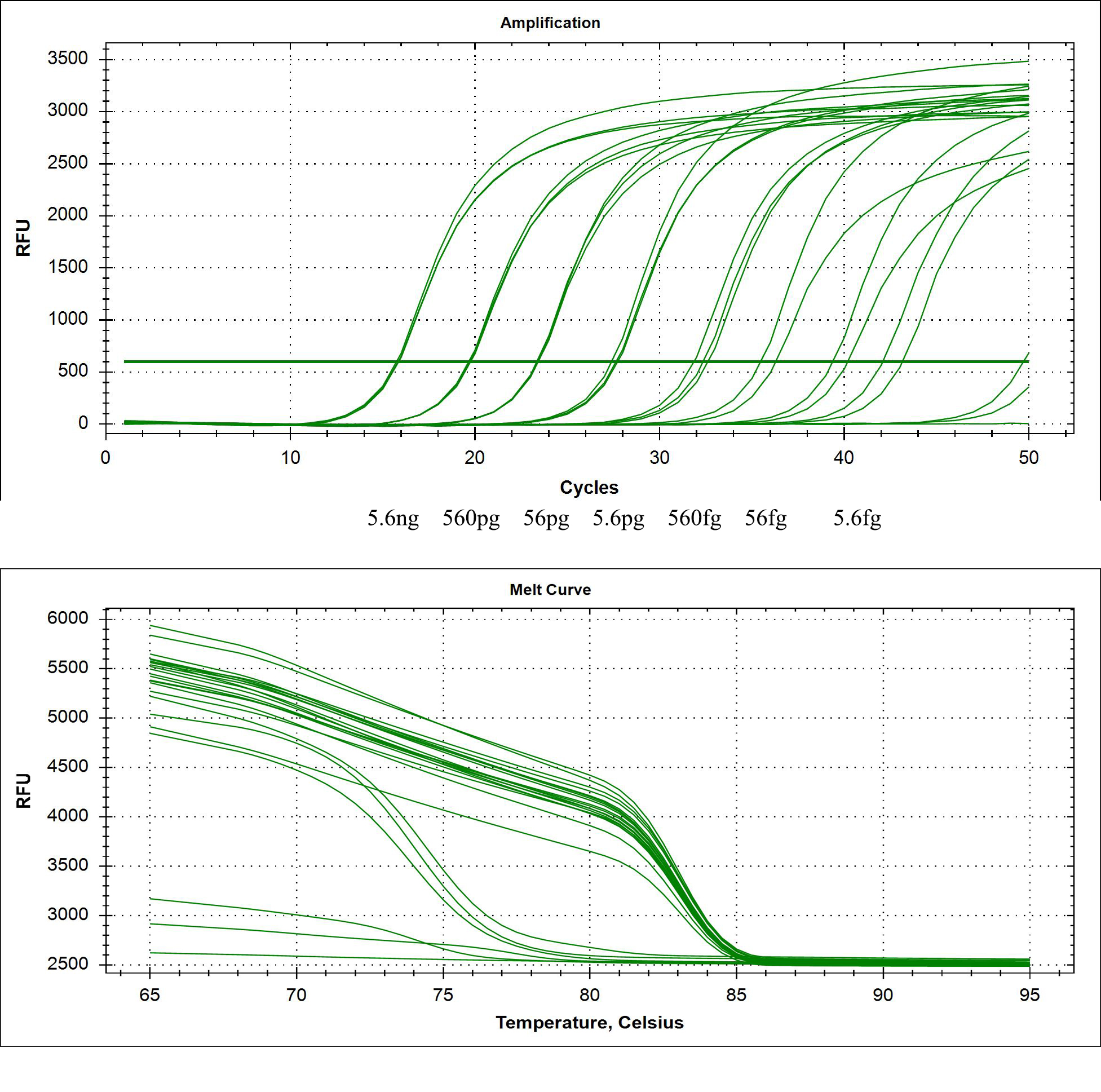

Supplement: Supplementary file 1 — Supplementary Figure S1. [file 41598_2021_87824_MOESM1_ESM.jpg]

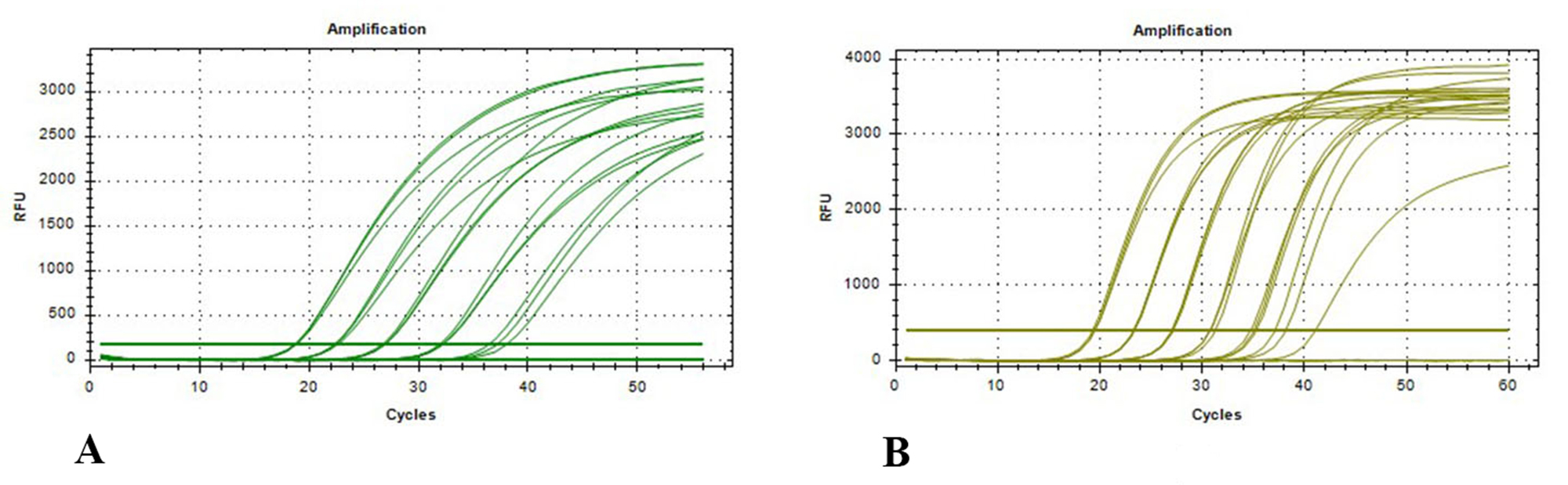

Supplement: Supplementary file 2 — Supplementary Figure S2. [file 41598_2021_87824_MOESM2_ESM.jpg]

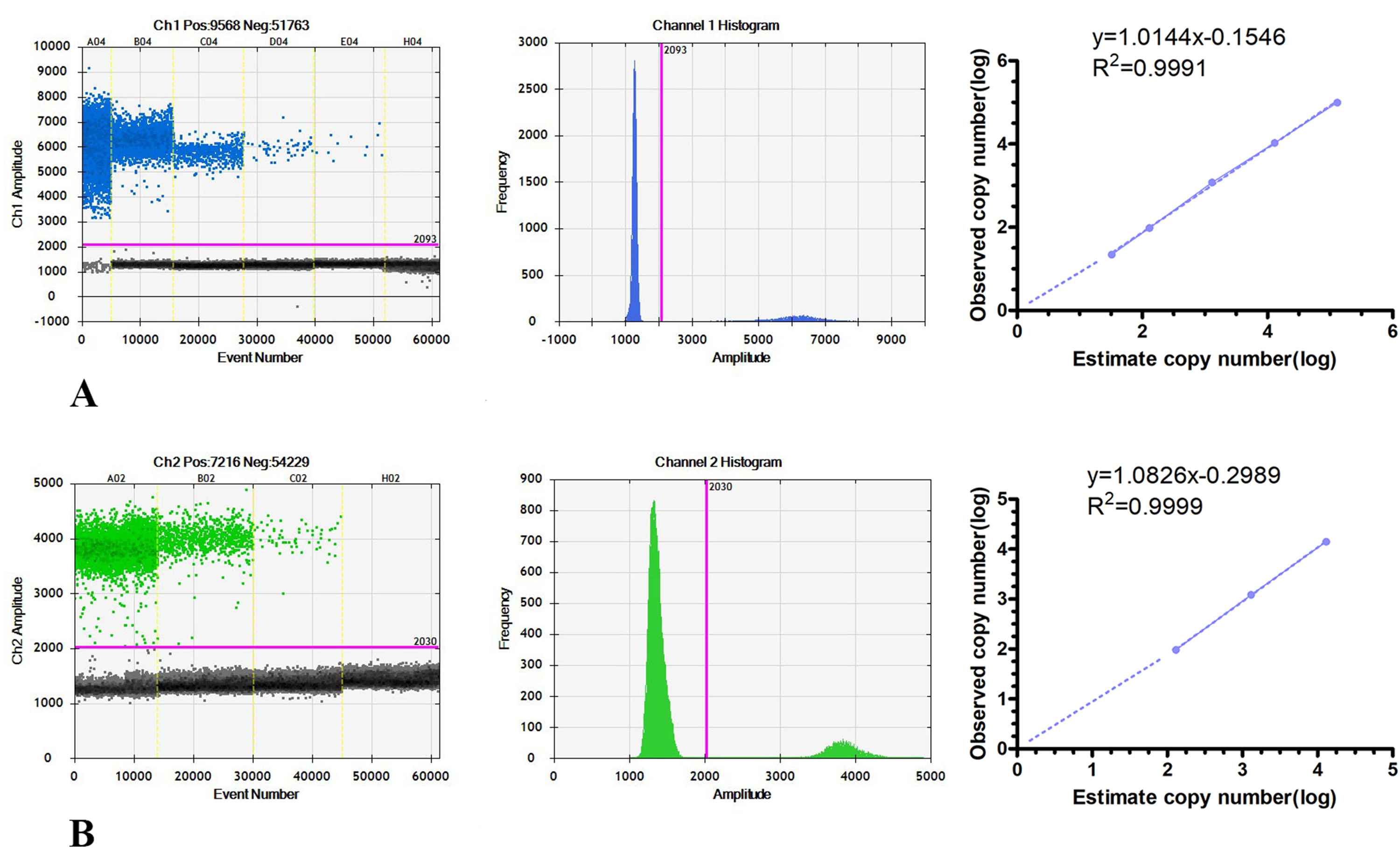

Supplement: Supplementary file 3 — Supplementary Figure S3. [file 41598_2021_87824_MOESM3_ESM.jpg]
