# Supplementary material for: Qualitative and quantitative detection of surgical pathogenic microorganisms Escherichia coli and Staphylococcus aureus based on ddPCR system
Source: Sci Rep. 2021 Apr 22;11:8771. doi: 10.1038/s41598-021-87824-5 (PMC8062461; doi:10.1038/s41598-021-87824-5)
Supplement: Supplementary file 4 — Supplementary Figure Legends [file 41598_2021_87824_MOESM4_ESM.docx]

**Figure S1.** **SYBR Green real-time quantitative PCR amplification curve and melt curve.** From 0 to 50 PCR cycles: the estimated copy numbers of *E. coli* ATCC25922 nucleic acid template were 1.3×10^6^, 1.3×10^5^, 1.3×10^4^, 1.3×10^3^, 1.3×10^2^, 13, 1.3 and NC.

**Figure S2. MGB probe real-time quantitative PCR amplification curve.** The designed MGB probes and primers for Escherichia coli and Staphylococcus aureus. A: amplification curve of Escherichia coli; B: amplification curve of Staphylococcus aureus.

**Figure S3. The dynamic range of the ddPCR-based assay.** Detecting the bacterial nucleic acid by ddPCR system, A: The 1D droplet spots of FAM fluorescence amplitude for eight detection sites for *E. coli-clin* (from A04 to H04: the estimated copy numbers of nucleic acid template were 1.3×10^5^, 1.3×10^4^, 1.3×10^3^, 1.3×10^2^, 32 and NC ) were performed for each detection site. Linear fitting lines are shown on the right B: The 1D droplet spots of VIC fluorescence amplitude for four detection sites for *Staphylococcus aureus* ATCC29213 ( from A01 to H01: the estimated copy numbers of nucleic acid template were 1.3×10^4^, 1.3×10^3^, 1.3×10^2^ and NC ) were performed for each detection site. Linear fitting lines are shown on the right.
