# Supplementary material for: Qualitative and quantitative detection of surgical pathogenic microorganisms Escherichia coli and Staphylococcus aureus based on ddPCR system
Source: Sci Rep. 2021 Apr 22;11:8771. doi: 10.1038/s41598-021-87824-5 (PMC8062461; doi:10.1038/s41598-021-87824-5)
Supplement: Supplementary file 5 — Supplementary Tables [file 41598_2021_87824_MOESM5_ESM.docx]

Table S1. Bacterial strains and sources

| Bacterial strain | source |
| --- | --- |
| E. coli standard strain  ATCC25922 | Department of Laboratory Medicine, Peking University People's Hospital |
| Escherichia coli clinical strain | Department of Laboratory Medicine, Peking University People's Hospital |
| Staphylococcus aureus standard strain ATCC25923 | Department of Laboratory Medicine, Peking University People's Hospital |
| Staphylococcus aureus standard strain ATCC25913 | Department of Laboratory Medicine, Peking University People's Hospital |

Table S2. Reagent name and manufacturer

| Reagent name | Manufacturer |
| --- | --- |
| Rapid plasmid mini kit | Beijing Tiangen Biotechnology Co., Ltd. |
| Qubit dsDNA HS Assay kit | Life Technologies |
| Cloning primer | Sangon Biotech(Shanghai)Co.,Ltd. |
| Specific amplification primer | Beijing Qingke Xinye Biotechnology Co., Ltd. |
| MGB probe | Invitrogen Trading(Shanghai)Co.,Ltd. |
| Glycerin | Sangon Biotech(Shanghai)Co.,Ltd. |
| 2×Taq Master Mix | Vazyme Biotech |
| ddPCRTM Supermix for probes（No dUTP） | Bio-rad |
| Droplet generation oil | Bio-rad |
| DG8 cartridge | Bio-rad |
| 96-well PCR plate | Eppendorf |
| Centrifuge tubes, pipette tips, PCR tubes | Axygen |

Table S3. Instrument name and manufacturer

| Instrument name | manufacturer |
| --- | --- |
| QX200™ Droplet Generator | Bio-rad |
| QX200™ Droplet Reader | Bio-rad |
| PX1TM PCR Plate Sealer | Bio-rad |
| Qubit3.0 fluorescence detector | Life Technologies |
| Desktop clean bench | Suzhou Sujie Purifying Equipment Co.,Ltd. |
| Pipette | Ependorf |
| Desktop small high-speed centrifuge | Ependorf |
| -20 ℃ low temperature refrigerator | Haier Group Co.,Ltd. |
| -80 ℃ ultra-low temperature refrigerator | Haier Group Co.,Ltd. |
| PCR instrument | Ependorf |
| Constant temperature incubator | Shanghai Zhenming Science Instrument Co.,Ltd. |
| Constant temperature oscillator | Suzhou Peiying Experimental Equipment Co., Ltd. |
| Water bath | Changzhou Dingfeng Instrument Manufacturing Co., Ltd. |

Table S4. E. coli primer and probe sequences

| Type Name | Sequence | Tm ℃ |
| --- | --- | --- |
| Forward Primer | TCACGCCGTATGTTATTG | 59 |
| Reverse Primer | GTCGGTAATCACCATTCC | 59 |
| MGB Probe | TGCCAGTTCAGTTCGTTGTTCAC | 70 |

Table S5. Primer and probe sequences of Staphylococcus aureus

| Type Name | Sequence | Tm ℃ |
| --- | --- | --- |
| Forward Primer | AGAGTTTGGTGCCTTTACAGATAGC | 59 |
| Reverse Primer | CTCATAGTGGCCAACAGTTTGC | 59 |
| MGB Probe | TGCCATACAGTCATTTC | 70 |

Table S6. ddPCR amplification system

| Ingredient | Volume(μL) |
| --- | --- |
| 1×ddPCRTM Supermix for probes (No dUTP) | 10 |
| Forward primer | 0.8 |
| Reverse primer | 0.8 |
| Probe | 0.4 |
| DNA template | 1 |
| Sterilized water | 7 |

Table S7. ddPCR amplification program

| Temperature(℃) | Time(s) | Remarks |
| --- | --- | --- |
| 95 | 600 | - |
| 94 | 30 | }40 cycles |
| 56 | 60 |  |
| 98 | 600 | - |
| 12 | ∞ | - |

Table S8. Detection of E. coli ATCC25922 nucleic acid template using SYBR Green real-time quantitative PCR system (n=3)

| The mass of nucleic acid template | 5.6ng | 560pg | 56pg | 5.6pg | 560fg | 56fg | 5.6fg | 0 |
| --- | --- | --- | --- | --- | --- | --- | --- | --- |
| Theoretical copy number | 1300000 | 130000 | 13000 | 1300 | 130 | 13 | 1.3 | 0 |
| Ct value | 16.67 | 20.50 | 24.22 | 28.16 | 32.30 | 35.91 | - | - |

Table S9. Detection of E. coli ATCC25922 nucleic acid template using the MGB probe real-time quantitative PCR system (n=3)

| The mass of nucleic acid template | 5.6ng | 560pg | 56pg | 5.6pg | 560fg | 56fg | 0 |
| --- | --- | --- | --- | --- | --- | --- | --- |
| Theoretical copy number | 1300000 | 130000 | 13000 | 1300 | 130 | 13 | 0 |
| Ct value | 18.81 | 22.49 | 26.93 | 32.16 | 37.27 | - | - |

Table S10. Detection of S. aureus ATCC25923 nucleic acid template using SYBR Green real-time quantitative PCR system (n=3)

| The mass of nucleic acid template | 3.1ng | 310pg | 31pg | 3.1pg | 310fg | 31fg | 0 |
| --- | --- | --- | --- | --- | --- | --- | --- |
| Theoretical copy number | 1300000 | 130000 | 13000 | 1300 | 130 | 13 | 0 |
| Ct value | 19.40 | 23.31 | 27.08 | 30.92 | 35.00 | - | - |

Table S11. Dynamic range of the ddPCR-based assay. Detecting the E. coli-clin nucleic acid template by ddPCR system

| Counts of bacterial nucleic acid template | 560pg | 56pg | 5.6pg | 1.4pg | 350fg | 0 |
| --- | --- | --- | --- | --- | --- | --- |
| Theoretical copy number | 130000 | 13000 | 1300 | 130 | 32.5 | 0 |
| Logarithmic transformation | 5.1139 | 4.1139 | 3.1139 | 2.1139 | 1.5119 | - |
| Actual number of copies | 99200 | 10600 | 1198 | 96 | 22 | 0 |
| Logarithmic transformation | 4.9965 | 4.0253 | 3.0784 | 1.9822 | 1.3424 | - |

Table S12. Dynamic range of the ddPCR-based assay. Detecting the Staphylococcus aureus ATCC29213 nucleic acid template by ddPCR system

| Counts of bacterial nucleic acid template | 31pg | 3.1pg | 310fg | 0 |
| --- | --- | --- | --- | --- |
| Theoretical copy number | 13000 | 1300 | 130 | 0 |
| Logarithmic transformation | 4.1139 | 3.1139 | 2.1139 | - |
| Actual number of copies | 14040 | 1220 | 96 | 0 |
| Logarithmic transformation | 4.1474 | 3.0864 | 1.9823 | - |
